# Dipeptidylpeptidase (DPP)-4 inhibitor therapy increases circulating levels of anti-inflammatory soluble frizzle receptor protein (sFRP)-5 which is decreased in severe COVID-19 disease

**DOI:** 10.1038/s41598-022-18354-x

**Published:** 2022-09-02

**Authors:** Juliane Brandes, Isabelle Zobel, Nathalie Rohmann, Kristina Schlicht, Corinna Geisler, Katharina Hartmann, Kathrin Türk, Witigo von Schönfels, Jan Beckmann, Florian Tran, Matthias Laudes

**Affiliations:** 1 Institute of Diabetes and Clinical Metabolic Research, University Medical Center Schleswig-Holstein (UKSH), Campus Kiel; Düsternbrooker Weg, 17, 24105 Kiel, Germany; 2 Department of General and Abdominal Surgery, University Medical Center Schleswig-Holstein (UKSH), Kiel, Germany; 3 Institute of Clinical Molecular Biology, University Medical Center Schleswig-Holstein (UKSH), Kiel, Germany; 4 Division of Endocrinology, Diabetes and Clinical Nutrition, Department of Medicine 1, University Medical Center Schleswig-Holstein (UKSH), Kiel, Germany; 5 Department of Internal Medicine I, University Medical Center Schleswig-Holstein, Campus Kiel, Kiel, Germany

**Keywords:** Immunology, Diseases

## Abstract

Obesity and type 2 diabetes (T2D) show an increased risk for a severe COVID-19 disease. Treatment with DPP4 inhibitor (DPP4i) results in reduced mortality and better clinical outcome. Here, we aimed to identify potential mechanisms for the observed DPP4i effect in COVID-19. Comparing T2D subjects with and without DPP4i treatment, we identified a significant increase of the anti-inflammatory adipokine sFRP5 in relation to DPP4 inhibition. sFRP5 is a specific antagonist to Wnt5a, a glycopeptide secreted by adipose tissue macrophages acting pro-inflammatory in various diseases. We therefore examined sFRP5 levels in patients hospitalised for severe COVID-19 and found significant lower levels compared to healthy controls. Since sFRP5 might consequently be a molecular link for the beneficial effects of DPP4i in COVID-19, we further aimed to identify the exact source of sFRP5 in adipose tissue on cellular level. We therefore isolated pre-adipocytes, mature adipocytes and macrophages from adipose tissue biopsies and performed western-blotting. Results showed a sFRP5 expression specifically in mature adipocytes of subcutaneous and omental adipose tissue. In summary, our data suggest that DPP4i increase serum levels of anti-inflammatory sFRP5 which might be beneficial in COVID-19, reflecting a state of sFRP5 deficiency.

## Introduction

The corona virus disease 2019 (COVID-19) is caused by severe acute respiratory syndrome coronavirus 2 (SARS-Cov-2). Subjects with type 2 diabetes (T2D) suffering from COVID-19 exhibit an increased risk for hospitalization and higher mortality in manifest COVID-19. In addition, recent studies showed that patients with T2D are at high risk for long term complications caused by SARS-Cov-2. Since 2020, various treatments have been tested to diminish symptoms and progress of this infectious disease^1,2^.

Dipeptidyl peptidase IV inhibitors (DPP4i), like Sitagliptin, are efficient antidiabetics revealing a two-or threefold increase of the gastro intestinal hormone glucagon-like peptide 1 (GLP-1) serum levels. GLP-1 induces 70% of the post-prandial insulin secretion and decreases glucagon serum levels under hyperglycaemic conditions. Due to the degradation of GLP-1 by DPP4, the bioavailability of GLP-1 is short-lived. DPP4 enzymes belong to the key regulators of incretin hormones^3,4^. Thus, DPP4i inhibit degradation of GLP-1 by DPP4 and ameliorate glucose dependent insulin response^5^.

Recent studies investigated beneficial effects of DPP4i in patients with T2D and concomitant severe infection with SARS-Cov-2. A multicentre, retrospective, case–control study from northern Italy, including 338 subjects with T2D and SARS-Cov-2, was performed. All subjects were hospitalized for severe COVID-19 and exhibited pneumonia. The cohort was stratified into subjects taking sitagliptin and subjects with standard of care. It was shown that patients under DPP4i treatment showed significantly enhanced clinical outcomes, reduced mortality and an increased number of clinical discharges compared to controls^6^.

To examine potential mechanisms mediating the anti-COVID-19 effects of DPP4i, we investigated the association between DPP4i intake and various clinical and biochemical markers as well as co-morbidities in T2D subjects in a subset of our cross-sectional Food Chain Plus (FoCus) cohort.

## Results

### DPP4i treatment is related to increased serum concentrations of the anti-inflammatory adipokine sFRP5 in human T2D subjects

**Figure 1:**
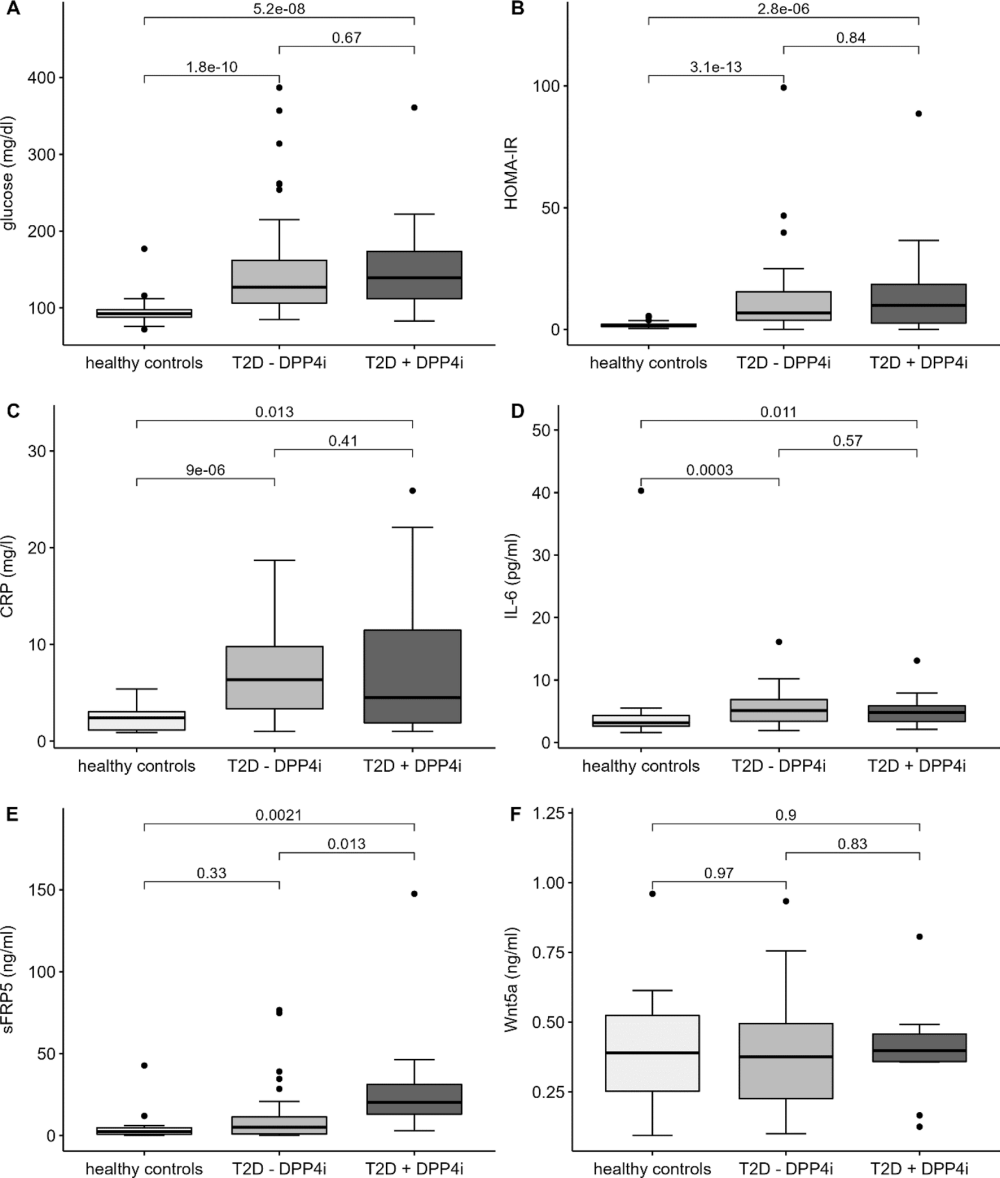
Differences of clinical parameters between FoCus subjects with T2D taking DPP4i (T2D + DPP4i), without DPP4i treatment (T2D-DPP4i) and healthy controls were investigated: Glucose (**A**), HOMA-IR (**B**), CRP (**C**), IL-6 (**D**) were significantly higher in diabetic subjects with and without DPP4i treatment compared to healthy controls, no differences between subjects with and without DPP4i treatment. (**E)** Diabetic subjects with DPP4i treatment showed significant higher levels of sFRP5 compared to diabetic subjects without DPP4i treatment and healthy controls. (**F)** Wnt5a levels showed no differences between groups. Wilcoxon test; statistical significance *p* < 0.05.

The aim of the present study was to examine, if DPP4i exert anti-inflammatory effects in treated T2D subjects which might explain the beneficial effects in Covid-19 patients. Therefore, differences in clinical and biochemical parameters between diabetic subjects with DPP4i treatment (T2D + DPP4i), without DPP4i treatment (T2D-DPP4i) and healthy controls from our FoCus cohort were investigated. While most of the tested variables were not different between the groups, a significant increase of the anti-inflammatory adipokine sFRP5 in diabetic subjects taking DPP4i compared to diabetic subjects without DPP4i treatment and healthy controls was found (*p* = 0.013; *p* = 0.0021, Fig. 1E).This is of interest, since the Wnt5a/sFRP5 system has been shown not only to be important in metabolic inflammation but also in severe bacterial sepsis^7^. In the present analysis, the pro-inflammatory counterpart, Wnt5a did not reveal significant differences between the groups (Fig. 1F). Besides sFRP5, parameters of glucose status: glucose and HOMA-IR (Fig. 1A,B), inflammatory markers: IL-6 and CRP (Fig. 1C,D) showed significant differences between healthy controls and diabetes subjects, but not between subjects with and without DPP4i treatment. Comorbidities, lipid profile, metabolites and anthropometry did not show significant differences between the diabetic subjects with and without DPP4i medication. Descriptive statistics and results of associations with clinical markers are shown in Tables 1 and 2.

**Table 1:** Basic characteristics of the FoCus subcohort stratified into subjects taking DPP4i (T2D + DPP4i), without DPP4i treatment (T2D-DPP4i) and healthy controls.

Basic characteristics of the FoCus subcohort				
Continuous variables	T2D + DPP4i (n = 23)	T2D-DPP4i (n = 46)	Healthy controls (n = 46)	*p*-value (T2D + DPP4i vs. T2D-DPP4i)
Age (years)	56 (± 11)	56 (± 11)	55 (± 11)	0.94
**Glucose status**				
Glucose (mg/dl)	139 (112; 174)	127 (106; 162)	93 (88; 98)	0.70
Insulin (mIU/l)	30 (13; 48)	22 (13; 35)	7 (5; 9)	0.44
HOMA Index	9.9 (2.6; 18.6)	6.7 (3.8; 15.5)	1.6 (1.1; 2.1)	0.84
**Lipid profile**				
Triglycerides (mg/dl)	158 (111; 249)	167 (126; 230)	81 (68; 107)	0.93
Lipoprotein a (mg/l)	329 (± 233)	239 (117; 436)	356 (142; 498)	0.82
Cholesterol (mmol/l)	4.1 (± 0.8)	4.5 (3.8; 5.5)	5.0 (± 0.9)	0.11
**Inflammatory markers**				
CRP (mg/l)	4.5 (1.9; 11.5)	6.4 (3.4; 9.8)	2.4 (1.2; 3.1)	0.41
IL-6 (pg/ml)	4.8 (3.3; 5.8)	5.1 (3.4; 6.9)	3.2 (2.6; 4.3)	0.57
Wnt5a (ng/ml)	0.4 (± 0.2)	0.4 (± 0.2)	0.4 (± 0.2)	0.83
SFRP5 (ng/ml)	**20.3 (13.1; 31.2)**	**5.1 (0.9; 11.4)**	**2.4 (0.8; 4.8)**	**0.01**
**Anthropometry**				
Weight (kg)	103 (93; 118)	124 (± 34)	74 (± 11)	0.13
Hip measure (cm)	122 (± 15)	124 (± 16)	103 (± 6)	0.58
Waist measure (cm)	118 (± 14)	120 (± 18)	89 (± 9)	0.65
BMI (weight/((height/100)^2)	38 (± 7)	41(± 10)	24 (± 2)	0.20
**Metabolites**				
Nicotinamid (µg/l)	14.9 (± 5.1)	16.0 (± 5.5)	16.7 (± 4.4)	0.58
Tryptophan (mg/dl)	1.8 (± 0.3)	1.7 (± 0.3)	1.3 (1.3; 1.7)	0.19

Values of normal distribution are shown as mean (standard deviation), values of non-normal distribution are shown as median (25th; 75th percentile); Subjects were matched by age and gender. Wilcoxon Test; statistical significance: *p* < 0.05.

Significant values are in bold.

**Table 2:** Basic characteristics of the FoCus subcohort stratified into subjects taking DPP4i (T2D + DPP4i), without DPP4i treatment (T2D-DPP4i) and healthy controls.

Categorical variasbles	T2D + DPP4i (n = 23)	T2D**-**DPP4i (n = 46)	Healthy controls (n = 46)	*p*-value (T2D + DPP4i vs. T2D-DPP4i)
**Sex**				
Male	12 (52%)	18 (39%)	27 (59%)	0.31
Female	11 (48%)	28 (61%)	19 (41%)	
**Inflammatory bowel disease**				
Yes	1 (4.3%)	3 (6.5%)	0 (0%)	0.73
No	22 (96%)	43 (93%)	46 (100%)	
**Cardian insufficiency**				
Yes	2 (9.5%)	6 (13%)	0 (0%)	0.67
No	19 (90%)	39 (87%)	46 (100%)	
**Apoplexy**				
Yes	2 (8.7%)	1 (2.2%)	0 (0%)	0.26
No	21 (91%)	44 (98%)	46 (100%)	
**Rheumatoid arthritis**				
Yes	1 (4.3%)	4 (9.5%)	0 (0%)	0.47
No	22 (96%)	38 (90%)	46 (100%)	
**Neuropathy**				
Yes	1 (4.8%)	7 (17%)	0 (0%)	0.19
No	20 (95%)	35 (83%)	46 (100%)	
**Periodontal disease**				
Yes	8 (35%)	17 (40%)	0 (0%)	0.71
No	15 (65%)	26 (60%)	46 (100%)	
**Liver disease**				
Yes	3 (13%)	8 (17%)	0 (0%)	0.65
No	20 (87%)	38 (83%)	46 (100%)	
**Myocardial infarction**				
Yes	3 (14%)	6 (13%)	0 (0%)	0.97
No	19 (86%)	40 (87%)	46 (100%)	
**High blood lipids**				
Yes	11 (50%)	24 (56%)	0 (0%)	0.66
No	11 (50%)	19 (44%)	46 (100%)	
**Hypertension**				
Yes	18 (78%)	35 (78%)	0 (0%)	0.97
No	5 (22%)	10 (22%)	46 (100%)	

Values of categorical variables are shown as n (%); Subjects were matched by age and gender. Wilcoxon Test; statistical significance: *p* < 0.05.

### Serum concentrations of sFRP5 are reduced in severe COVID-19 disease

**Figure 2:**
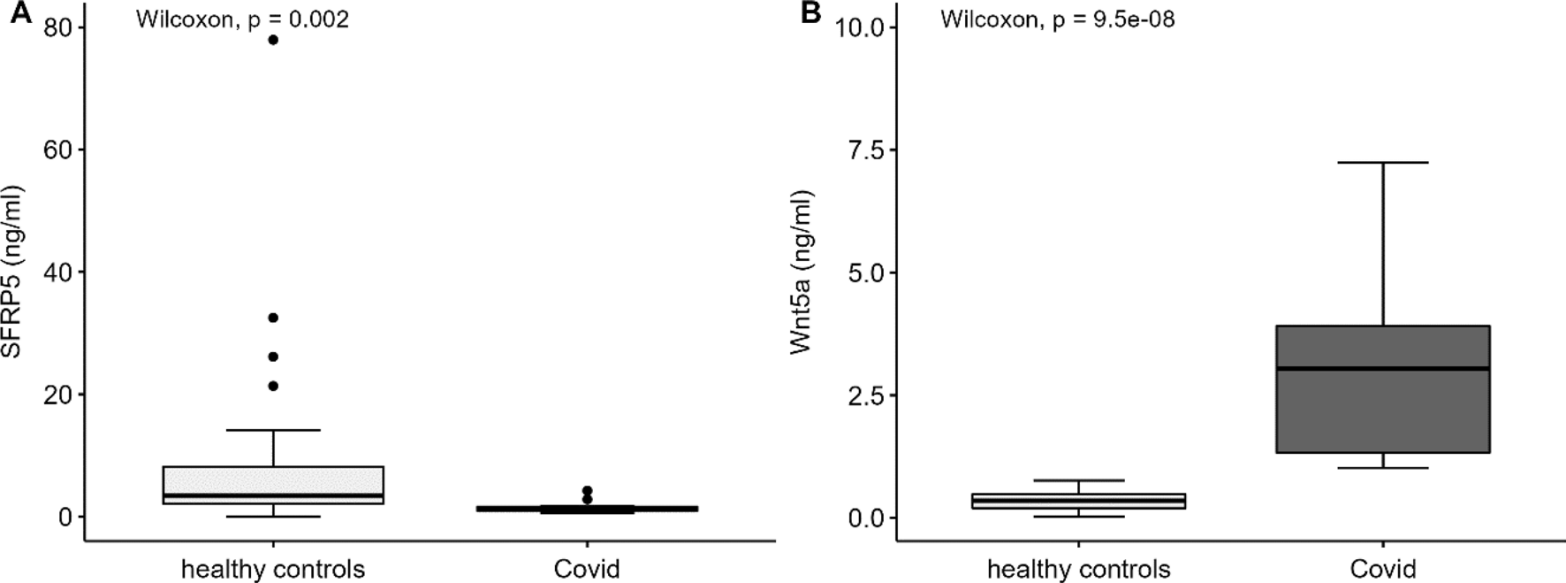
Differences in sFRP5 and Wnt5a levels between patients from ICU cohort with severe Covid-19 infection (cases) and healthy controls from FoCus cohort. (**A**) Subjects with Covid-19 disease showed significant lower sFRP5 levels compared to healthy controls. (**B**) Levels of Wnt5a were significantly increased in cases compared to healthy controls. Wilcoxon Test; statistical significance *p* < 0.05.

Having found that anti-inflammatory sFRP5 is increased in DPP4i treated T2D subjects we next examined, if sFRP5 and Wnt5a are altered in COVID-19 patients. These examinations were performed in a cohort of 17 patients hospitalised on intensive care unit for severe COVID-19 and compared to healthy controls from our FoCus cohort. Table 3 displays basic characteristics of the Covid-19 subjects from the ICU-cohort and healthy controls from the FoCus cohort. Of interest, we found that subjects with COVID-19 revealed significant lower sFRP5 levels compared to healthy controls (*p* < 0.01, Fig. 2A). Moreover, Wnt5a levels were significantly increased in COVID-19 patients compared to healthy controls (*p* < 0.001, Fig. 2B).

**Table 3:** Basic characteristics of the Covid-19 subjects from the ICU cohort and healthy controls from the FoCus cohort.

Basic characteristics of covid-19 subjects and healthy controls		
Continuous variables	Covid-19 subjects n = 17	Healthy controls n = 34
Age (years)	57 (40; 73)	50 (± 16)
BMI	–	25 (± 3)
sFRP5 (ng/ml)	1.56 (1.34; 1.65)	3.25 (1.56; 8.91)
Wnt5a (ng/ml)	3.05 (1.50; 3.98)	0.36 (± 0.19)
Granulocytes (cells/l)	7.12 (6.25; 14.90)	–
Leukocytes (× 10^9^/l)	11 (6; 19)	–
Lymphocytes (cells/l)	1.10 (1.04; 1.15)	–
Platelets (cells/l)	220 (101; 278)	–
GOT (U/l)	94 (58; 189)	–
GGT (U/l)	304 (182; 575)	–
IL-6 (pg/ml)	43.05 (19.55; 61.65)	3.1 (2.40; 3.75)
CRP (mg/l)	69 (34.20; 98.10)	1.40 (1.20; 2.50)
Creatinine (µmol/l)	106 (52; 131)	–
D-Dimer (mg/l FEU)	2.9 (1.8; 5.7)	–
**Categorical variables**		
Gender: Male/Female	12 (71%)/5 (29%)	25 (74%)/9 (26%)
Hypertension: Yes/No	9 (53%)/8 (47%)	–
Hyperlipidemia: Yes/No	4 (24%)/13 (76%)	–
Type 2 Diabetes: Yes/No	6 (35%)/11 (65%)	–

Values of normal distribution are shown as mean (standard deviation), values of non-normal distribution are shown as median (25th; 75th percentile); Subjects were matched by age and gender.

### sFRP5 is specifically expressed in mature adipocytes

So far, we found that DPP-4 inhibitor treatment is associated with increased sFRP5 serum concentrations and that COVID-19 is related to sFRP5 deficiency. sFRP5 is known to be an adipokine. In a next set of experiments, we aimed to examine, (1) which specific cell type in adipose tissue is important for sFRP5 synthesis and release in order to identify the potential DPP4i cellular target and (2) whether sFRP5 is differentially expressed in subcutaneous (SAT) versus visceral adipose tissue (VAT), since it is known that inflammatory reactions are more closely linked to visceral adipose tissue. To this end we isolated pre-adipocytes, mature adipocytes and macrophages from adipose tissue biopsies from *n* = 100 human subjects undergoing elective abdominal surgery (mainly bariatric surgery). The basic characteristics of the adipose tissue biopsy cohort are given in Table 4.

**Table 4:** Basic characteristics of the adipose tissue study cohort.

Basic characteristics of adipose tissue study cohort			
Variables	Men	Women	All subjects
n	33	67	100
Age (years)	56** (46; 64)	45** (37; 53)	48 (39; 57)
Weight (kg)	153 (126; 166)	144 (128; 159)	148 (127; 164)
Height (cm)	180** (175; 185)	171** (165; 174)	173 (167; 178)
BMI (kg/m^2^)	47 (39; 54)	50 (45; 54)	49 (43; 54)
Visceral sample size [g]	2.96* (1.52; 6.08)	2.07* (1.60; 2.94)	2.20 (1.59; 3.84)
Subcutaneous sample size [g]	1.43 (1.07; 1.96)	1.69 (1.22; 2.13)	1.58 (1.12; 2.09)

Values are shown as median (25th and 75th percentiles); Mann–Whitney-U-test (*p < 0.05; **p < 0.01) (statistical significance *p* < 0.05).

**Figure 3:**
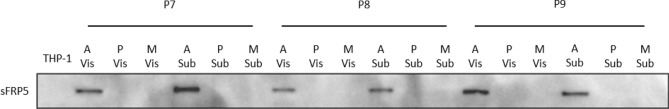
Investigation of sFRP5 expression in different cell types of the adipose tissue study cohort: Western blot: sFRP5 expression was analysed in THP-1 control, visceral and subcutaneous adipocytes, pre-adipocytes and macrophages from three representative samples (patient P7, P8, P9). Only visceral and subcutaneous adipocytes showed sFRP5 expression.

Protein expression of sFRP5 was investigated by western blotting. sFRP5 was not detectable in pre-adipocytes or macrophages. In contrast, mature adipocytes revealed a significant sFRP5 expression (Fig. 3) suggesting sFRP5 is an adipokine specifically secreted by fully differentiated adipocytes in humans.

Of interest, although inflammation is more linked to VAT, sFRP5 expression was found in both, mature adipocytes from subcutaneous and visceral adipose tissue biopsies with no significant difference.

## Discussion

Patients with T2D and severe COVID-19 disease showed better clinical outcomes with intake of the DPP4i sitagliptin^6^. sDPP4 activity was described to be elevated during infections with hepatitis C virus or Epstein-Barr virus^8^. DPP4 cleaves the pro-inflammatory chemokine CXCL10 leading to the short NH2-terminal truncated CXCL10 form which is positively associated with hepatitis C virus infection^9–11^. Treatment with DPP4i reduced cleavage of CXCL10^12^ which points out that sDPP4i exhibit more therapeutic functions than increasing GLP-1 levels.

To examine further potential mechanisms of the anti-viral/anti-inflammatory DPP4i activity, we compared T2D patients treated with DPP4i (*n* = 23), without DPP4i (*n* = 46) and healthy controls (*n* = 46) on various clinical and biochemical levels. Significant higher levels of the Wnt5a inhibitor sFRP5 in T2D subjects with DPP4i treatment compared to T2D subjects without DPP4i treatment and healthy controls were found. Metabolic markers such as glucose and HOMA-IR and the inflammatory parameters CRP and IL-6 showed significant higher levels in diabetic subjects with and without DPP4i treatment compared to healthy controls. However, diabetic subjects with and without DPP4i medication had no differences in these clinical parameters which points out the independent elevation of SFRP5 by DPP4 inhibition. Our previous studies showed that the pro-inflammatory Wnt5a/anti-inflammatory sFRP5 system is closely related to various acute (e.g. sepsis^7^) as well as chronic (e.g. psoriasis^13^) inflammatory diseases and is also important in the context of metabolic inflammation in obesity and type 2 diabetes. Thereby, obese subjects reveal reduced concentrations of anti-inflammatory sFRP5 and increased levels of Wnt5a^14,15^.

In our previous clinical study in human subjects suffering from bacterial sepsis we observed stable (not reduced) sFRP5 concentrations^7^. This is of interest, since in the present analysis sFRP5 concentrations were reduced in severe COVID-19 disease, suggesting differences of sFRP5 in bacterial versus COVID-19 acute systemic inflammation.

Wnt5a is a glycoprotein and displays a regulator of the innate immune response by reinforcing low-grade inflammation. It is secreted by pro-inflammatory macrophages which infiltrate adipose tissue. Previous investigations of our working group showed in a FoCus subcohort of *n* = 896 cross-sectional subjects a positive association of Wnt5a with the clinical markers IL-6 and triglycerides. Furthermore, subjects with T2D exhibited high levels of Wnt5a which were positively correlated with fasting plasma glucose levels^15^. sFRP5 is a soluble Wnt5a receptor and inhibits pro-inflammatory effects of Wnt5a by sequestering it extracellularly. Accordingly, sFRP5 has been indicated as potential therapeutic target in metabolic inflammation^7,15,16^.

Having found DPP4i treatment to be related with increased sFRP5 serum levels, we specifically analysed differences of serum sFRP5 levels between subjects with severe COVID-19 disease and healthy Focus controls. We observed significant lower sFRP5 levels in COVID-19 patients compared to healthy controls, while Wnt5a levels were significantly increased in COVID-19 patients. Of interest, Choi et al. recently also showed significantly increased Wnt5a serum levels in severe COVID-19 infection^17^. Hence, results point towards the activation of wnt signalling pathway in COVID-19, that might be even more pronounced in severe inflammation resulting from SARS-CoV-2 rather than classical bacterial septic infection due to the additional reduction of anti-inflammatory sFRP5 in COVID-19 but not in bacterial sepsis. Furthermore, it might be of interest how sFRP5/wnt5a levels act in subjects with mild COVID-19 to prevent a possible more severe course of disease or post covid-19 symptoms.

To further investigate sFRP5 characteristics in metabolic inflammation, we analysed differential expression of sFRP5 on a cellular level in pre-adipocytes, adipocytes and macrophages. Results showed sFRP5 expression specifically in visceral and subcutaneous mature adipocytes while for pre-adipocytes and macrophages, no sFRP5 expression was recorded. These results confirm the findings of other working groups^18,19^ which indicates that compared to Wnt5a, sFRP5 reflects a specific marker of mature adipocytes. Furthermore, Wang et al. showed that sFRP5 expression showed higher levels in VAT from obese patients compared to lean controls^18^. These findings contradict the assumption that sFRP5 is only expressed in healthy adipocytes and reveals decreased levels in obesity^20^. It is also to be mentioned that sFRP5 is not only expressed in adipose tissue but also in pancreatic islets in which sFRP5 and Wnt signalling were shown to be regulators of *β*-cell proliferation^21^. Since it was observed that a SARS-Cov-2 infection can induce *β*-cell damage and therefore reinforce the development of T2D^22^, it is of importance to investigate the effects of DPP4i not only in the adipose tissue but also in pancreatic islets.

Thus, the results of our present study together with our previous results in obese subjects^15^ might explain obesity being a risk factor for severe disease courses of COVID-19, since sFRP5 levels are lower in both, COVID-19 and severe obesity. However, a limitation of this analysis must be mentioned that the BMI of Covid-19 subjects could not be determined because they were in intensive care. Only two subjects were diagnosed as obese.

Besides Wnt5a scavenging, the specific mechanisms of sFRP5 are still barely investigated. According to its anti-inflammatory properties, Carstensen-Kirberg et al. observed that sFRP5 decreased levels of Il-6 and NF-κB phosphorylation in TNFα treated human adipocytes^23^, further suggesting anti-inflammatory activities.

In summary, our data suggest that the release of the anti-inflammatory adipokine sFRP5 from mature adipocytes in humans is induced by DPP4i which might compensate the sFRP5 deficiency found in COVID-19, but not in bacterial sepsis. In addition, it might be speculated that elevated levels of sFRP5 might be an indication for a better clinical outcome of COVID-19 in obese as well as T2D patients, which could be interesting to examine in future biomarker studies.

## Methods

### Clinical cohorts used in the study

The Food Chain Plus (FoCus) is a cross-sectional, population-based cohort and included 511 subjects from the obesity outpatient clinic (Department of Internal Medicine I, University Medical Center Schleswig–Holstein, Campus Kiel, Germany) and 1326 people from the regional registration offices as cross-sectional control subjects. The intermediate care unit (ICU) cohort from the institute of clinical molecular biology (IKMB, University Medical Center Schleswig–Holstein, Campus Kiel, Germany) consisted of 25 Covid-19 patients including recall subjects. Adipose tissue biopsies were obtained from 100 subjects (Institute for experimental tumor research, University Medical Center Schleswig–Holstein, Campus Kiel, Germany) undergoing elective abdominal surgery, most of them bariatric therapy. All studies were approved by the local ethics committee (Christian-Albrechts University in Kiel) and all patients signed the informed consent.

We generally confirm that all experiments were performed in accordance with relevant guidelines and regulations.

### Anthropometric characteristics and blood pressure measurements

Weight and height were measured by digital scales and a stadiometer. They were used to calculate BMI [weight (kg) divided by height (m)2]. Waist circumference was measured at the approximate midpoint between the lower margin of the last palpable rib and the top of the iliac crest, according to the World Health Organization^24^.

### Biochemical analysis

After an overnight fast, blood samples from Focus cohort were obtained by venipuncture. The central laboratory (UKSH, Kiel) analysed the following parameters: C-reactive protein (CRP) by immunoturbidimetry (Hitachi Modular; Roche), fasting glucose by glucose-hexokinase-ultraviolet test (Hitachi Modular), IL-6 and fasting insulin by electrochemiluminescence immunoassay (Elecsys System 2010; Roche), and triglycerides by enzymatic test (Hitachi Modular; Roche)^25^. Homeostatic model assessment of insulin resistance (HOMA-IR) was calculated as fasting glucose (milligrams per deciliter) multiplied by fasting insulin (microunits per milliliter) and that product divided by 405 to determine insulin resistance. Protein concentrations of sFRP5 and Wnt5a were measured using the following ELISA kits: Wnt5a (MBS162566; sample dilution: 1:2 or 1:3 with PBS depending on available amount of serum; analytic sensitivity < 0.059 ng/mL; MyBioScource; San Diego, US), sFRP5 (SEC842Hu; analytic sensitivity < 0.58 ng/mL; Cloud-Clone Corp; Hölzel Diagnostika, Cologne, Germany). Investigations were performed according to the manufacturer’s instructions. Variables of comorbidities and intake of DPP4i were evaluated by a questionnaire.

### Isolation of adipocytes, pre-adipocytes and macrophages from human adipose tissue biopsies

Subcutaneous and visceral adipose tissue (SAT, VAT) biopsies from bariatric surgeries or other surgeries (lean controls) were mechanically cut to a homogeneous mixture and digested with collagenase type II (Sigma Aldrich; Darmstadt, Germany) for 1 h at 37 °C in a shaking incubator. After digestion the samples were filtered with a sieve (355 µm; 280 µm) to separate cells from undigested tissue. Afterwards, the samples were centrifuged (1000 rpm; 5 min). Adipocytes from the upper phase were pipetted in a reaction vessel and frozen at − 80 °C. Remaining supernatants were removed and the cell pellets were resuspended in 5 mL red cell lysis buffer and incubated for 5 min at room temperature. After centrifugation, cell pellets were resuspended in 10 mL isotonic NaCl solution (Berlin Chemie AG, Germany). Up to 1 × 10^7^ cells were resuspended in 80 µl MACS-buffer and 20 µl CD14 magnetic micro-beads (Miltenyi Biotec; Bergisch-Gladbach, Germany) and incubated for 15 min at 4 °C. Afterwards, cells were washed with 1 mL MACS-buffer and centrifuged (300xg for 10 min). Cells were resuspended in 500 µL MACS-buffer. For magnetic separation of macrophages and pre-adipocytes, MACS MS columns with pre-separation filters and a magnetic MiniMACS Separator (Miltenyi Biotec; Bergisch-Gladbach, Germany) was used. Macrophages and pre-adipocytes samples were centrifuged (200xg; 12 min), resuspended in 200 µl APL lysis buffer and frozen at − 80 °C.

### Preparation of total protein from mature adipocytes, pre-adipocytes and macrophages

Protein was isolated from primary human pre-adipocytes and macrophages using the AllPrep Protein Kit (Qiagen; Hilden, Germany) following the manufacturer’s instructions. Total protein of human adipocytes was isolated using the Minute Total Protein Extraction Kit for Adipose Tissue/Cultured Adipocytes (Invent Biotechnologies, MN, USA). Protein concentrations were measured with the spectrophotometer Nanodrop one (Thermo Fisher Scientific, Inc., MA, USA). Samples, containing low protein were desalted with Zeba Spin Columns 0.5 mL 7K MWCO (Thermo Fisher Scientific, Inc., MA, USA) following the manufacturer`s instructions. Samples were dried overnight at room temperature using the Speed-Vac machine concentrator 5301 (Eppendorf; Hamburg, Germany) or at − 80 °C using the lyophilisation machine Alpha 2-4 LSC (Christ; Osterode am Harz, Germany). A complete set of proteins (from visceral and subcutaneous adipocytes, pre-adipocytes and macrophages) could be isolated from *n* = 70 patients which were used for further analysis. Since general loading control markers such as *β*-actin, *α*-tubulin and GAPDH were not suitable, the membranes were stained with Ponceau-S solution.

### Western-blotting

A Bradford assay was conducted and the protein assay dye reagent (BIO-RAD; Ca, USA) was used. Protein (30 µg per sample) was added to 4 × loading buffer (bromophenol blue and Laemmli buffer), heated to 95 °C for 5 min, and separated on a 12.5% SDS-PAGE gel. Afterwards, proteins were transferred to polyvinylidene fluoride membranes (Carl Roth, Karlsruhe, Germany). The following antibodies were used according to the instructions of the manufacturer: sFRP5 (Santa Cruz Biotechnology; Dallas, Texas; USA) and secondary antibody anti-rabbit (Cell signalling; Danvers, Massachusetts, USA). Membranes were incubated with SuperSignal West Femto Maximum Sensitivity Substrate (Thermo Scientific; Waltham, Massachusetts, USA) for 5 min at room temperature and the signals were detected with the Molecular Imager ChemiDoc XRS+ (Bio Rad; Hercules, California, USA).

### Statistical analysis

Statistical analysis was performed using open source R software and SPSS Statistics Software Version 24. Statistical significance was set at *p* < 0.05.

From FoCus cohort, a subcohort of patients was divided into subjects with type 2 diabetes taking DPP4i (*n* = 23; “T2D + DPP4i”), without DPP4i treatment (*n* = 46; “T2D-DPP4i”) and healthy subjects (*n* = 46; healthy controls). Groups were matched by age and gender. Continuous variables were tested for normal distribution using the Shapiro–Wilk test. To determine between-group differences, Wilcoxon rank sum test was performed. Categorical variables are shown as absolute numbers and percentages. x^2^ test and Fisher test were used for between-group analyses.

The ICU cohort contained 25 subjects. Since recalls were excluded, 17 Covid-19 subjects of the ICU cohort were selected and matched by gender and age with healthy controls of FoCus cohort (*n* = 34). Since Covid-19 subjects were in intensive care, the BMI could not be determined. However, 2 ICU subjects were diagnosed as obese. Healthy controls had a BMI lower than 25. sFRP5 and Wnt5a differences between groups were determined using Wilcoxon rank sum test.

For descriptive statistics, variables of adipose tissue cohort were tested for normal distribution using the Shapiro–Wilk test and are expressed as median (IQR) according to non-normal distribution. Mann–Whitney U test were used to determine group-differences.

## Supplementary Information

Supplementary Information.

## Data Availability

The datasets generated and/or analysed during the current study are available from the corresponding author on reasonable request.
