# Supplementary material for: Dipeptidylpeptidase (DPP)-4 inhibitor therapy increases circulating levels of anti-inflammatory soluble frizzle receptor protein (sFRP)-5 which is decreased in severe COVID-19 disease
Source: Sci Rep. 2022 Sep 2;12:14935. doi: 10.1038/s41598-022-18354-x (PMC9437412; doi:10.1038/s41598-022-18354-x)
Supplement: Supplementary file 1 — Supplementary Information. [file 41598_2022_18354_MOESM1_ESM.pdf]

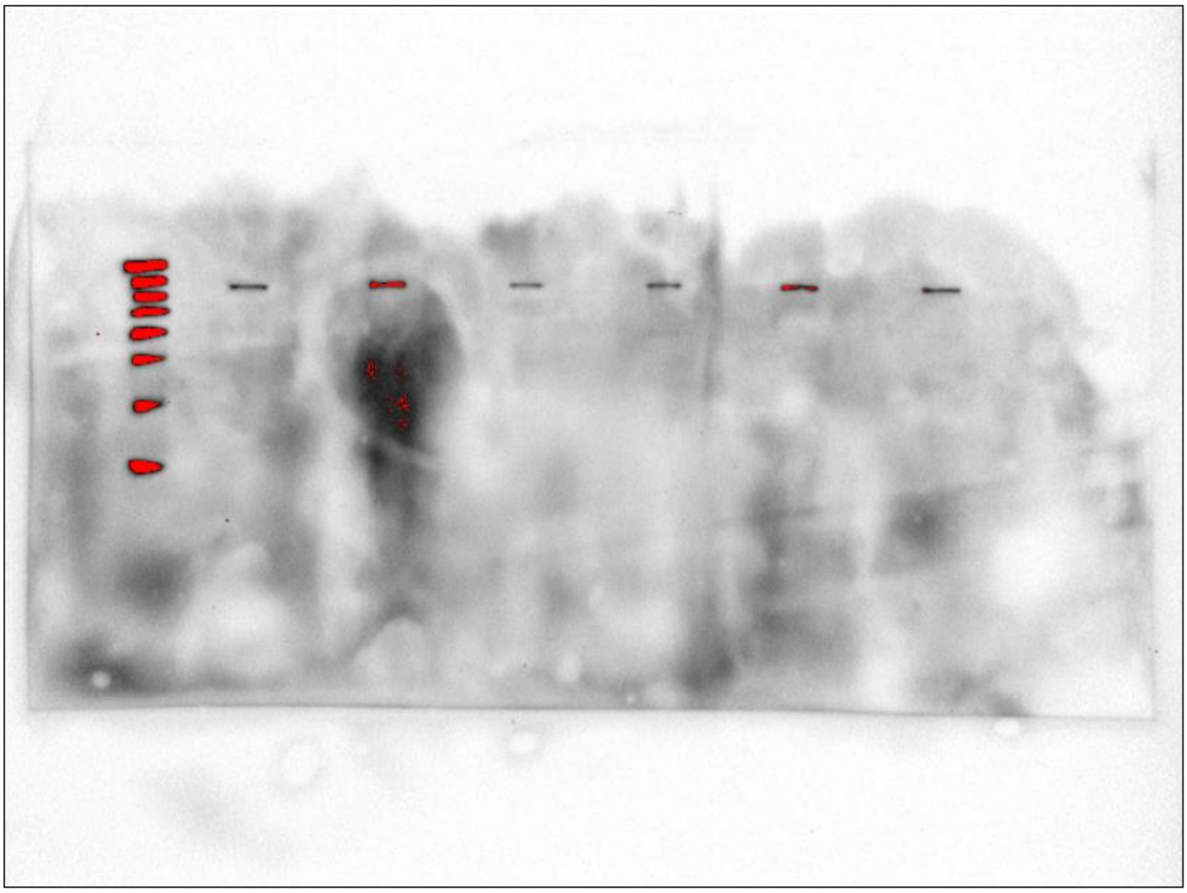

***Figure S1: Expression of sFRP5 in different cell types in adipose tissue**: Shown is the full image of the western bot membrane of Figure 3*
